# Hydrogel Actuators and Sensors for Biomedical Soft Robots: Brief Overview with Impending Challenges

**DOI:** 10.3390/biomimetics3030015

**Published:** 2018-07-10

**Authors:** Hritwick Banerjee, Mohamed Suhail, Hongliang Ren

**Affiliations:** 1 Department of Biomedical Engineering, Faculty of Engineering, 4 Engineering Drive 3, National University of Singapore, Singapore 117583, Singapore; biehb@nus.edu.sg (H.B.); msuhail1997@gmail.com (M.S.); 2 Singapore Institute for Neurotechnology (SINAPSE), Centre for Life Sciences, National University of Singapore, 28 Medical Drive, #05-COR, Singapore 117456, Singapore; 3 Department of Mechancial Engineering, National Institute of Technology, Tiruchirappalli, Tamil Nadu 620015, India; 4 National University of Singapore (Suzhou) Research Institute (NUSRI), 377 Lin Quan Street, Suzhou Industrial Park, Suzhou 215123, China

**Keywords:** hydrogel robots, biomedical sensors, soft actuators, stimuli-responsive hydrogels

## Abstract

There are numerous developments taking place in the field of biorobotics, and one such recent breakthrough is the implementation of soft robots—a pathway to mimic nature’s organic parts for research purposes and in minimally invasive surgeries as a result of their shape-morphing and adaptable features. Hydrogels (biocompatible, biodegradable materials that are used in designing soft robots and sensor integration), have come into demand because of their beneficial properties, such as high water content, flexibility, and multi-faceted advantages particularly in targeted drug delivery, surgery and biorobotics. We illustrate in this review article the different types of biomedical sensors and actuators for which a hydrogel acts as an active primary material, and we elucidate their limitations and the future scope of this material in the nexus of similar biomedical avenues.

## 1. Introduction

Hydrogels are in the family of three-dimensional (3D) polymeric materials for which the bulk of the matrix consists of water (typically 80–90 wt %) [1,2]. The polymers cross-link with each other to form a network, and water molecules ingress into the network, swelling the gel [3,4,5] while being responsive to different external stimuli, such as temperature, light, pH, ionic strength, magnetism, and pressure. Additionally, these matrices are mechanically compliant [5,6] and promote safer interactions with surrounding biological tissues [7]. The mechanics of these hydrogel matrices can further be tuned and optimized by changing the compositions in the matrix [6], tailoring to the specific clinical needs [8,9]. As a result of their high water content, hydrogels also possess excellent diffusive properties and thus are widely used in drug delivery [10,11,12,13], regenerative medicine [14,15], tissue engineering [16,17,18,19], artificial organs [20,21], and related fields [9,22,23]. The shape and form of the gel can be customized via molding and fabrication techniques as discussed in the later sections of this review article [24]. This allows the hydrogel components to mimic biotissues and organs as well as design actuators and sensors for various biomedical applications [25]. While hydrogels and gels, in general, have attracted much attention in soft robotics and drug delivery research, review articles on hydrogel materials are mostly targeted for the chemical and polymer research community. Review articles on gel-based designs in robotic research benefit the community by addressing the necessary design parameters for robotic research that are less noted in the chemical and polymer community. Here, we restrict our focus only to hydrogel-based actuators and sensors mostly from an enhanced-mechanical-characteristics standpoint, which, in the long run, will be applied in diversified biomedical applications.

In order to manufacture soft, flexible, yet resilient and robust soft sensors and actuators, materials scientists and roboticists are facing a great challenge in characterizing new polymers, biomimetic materials from a biocompatible, degradable, and environmentally friendly point of view [26,27].

In most of the cases, when there is a need to use a flexible, synthetic polymer for making shape-adaptable, deformable robots, a silicone elastomer is used extensively because of its simple, rapid fabrication procedure [28,29]. Silicone elastomers in general belong to a family of natural/synthetic polymers having robust elastic properties, for example, rubber, polysiloxane made of chains of silicone, and oxygen atoms [30,31,32]. Although silicone elastomers have been used extensively in the field of soft sensing and biomedical soft robots, problems such as biocompatibility issues [33,34], an inability to scale down to the micro/nano regime as a result of higher viscosity (i.e., Ecoflex 00-30 viscosity 3000 cps, Smooth-On) [35], hydrophobicity, and toxicity threats [36] make them unsuitable in many biomedical applications. On the other hand, because of the innate customizability and biocompatibility of hydrogels, scientists have been leaning more towards their use in recent times; prototype scaling down has high feasible choices and thus is used in biomaterials with various biological cell interactions [7,37,38]. Therefore, it is beneficial to combine the unique properties of hydrogels and elastomers into a composite biomaterial through methods such as free-radical cross-linking copolymerization [20,39,40,41,42,43,44,45,46,47,48,49,50]. In Table 1, we show some key differences that have led hydrogels to be used as opposed to their potential counterparts, particularly in biological environments.

In a conscious attempt not to engulf readers, the paper illustrates some definitions of key terms used in the subsequent sections. For example, “rigidity” is here defined as the property of a material resisting distortion in any direction (isotropic) and everywhere (homogeneous) while an external load is applied [51]. In contrast, “stiffness” means a material can exhibit directional motion while there is a unidirectional load (tension), while for tortion and twisting it may easily deform. Thus, stiffness is defined as the rigidity of a material by virtue of which it can resist deformation up to an extent posed by the external load. Likewise, “elasticity” demonstrates a property of the material for which the nature of the deformation is reversible and the energy principle remains valid when the external/internal load is diminished. In the subsequent sections (Section 2 and Section 3), we illustrate some specific case studies in the realm of actuators and sensors that can potentially revolutionize biomedical applications in the near future. As a part of this mechanical rigidity–stability–deformation standpoint, we outline our discussion on the basis of the various natures of gels and their characteristics in Section 4, and we quantify these in Section 5.

## 2. Hydrogel-Based Soft Actuators

The principle of working with hydrogel–polymer-based soft actuators is that the hydrogel material has the capability to swell/shrink depending on the amount of water present in it, which allows us to generate various actuations and motions. This water content is responsive and sensitive to various external stimuli, such as heat, electricity, magnetism, strain, pH, molecular interactions, ionic strength, and so on. Hence, there is a wide scope of applications in different fields for hydrogel-based actuators, despite that they have the limitation of acting only in aqueous media [52]. In Table 2, we show the state-of-the-art actuation regime for comparison with the hydrogel-based actuation principle.

**Figure 1:**
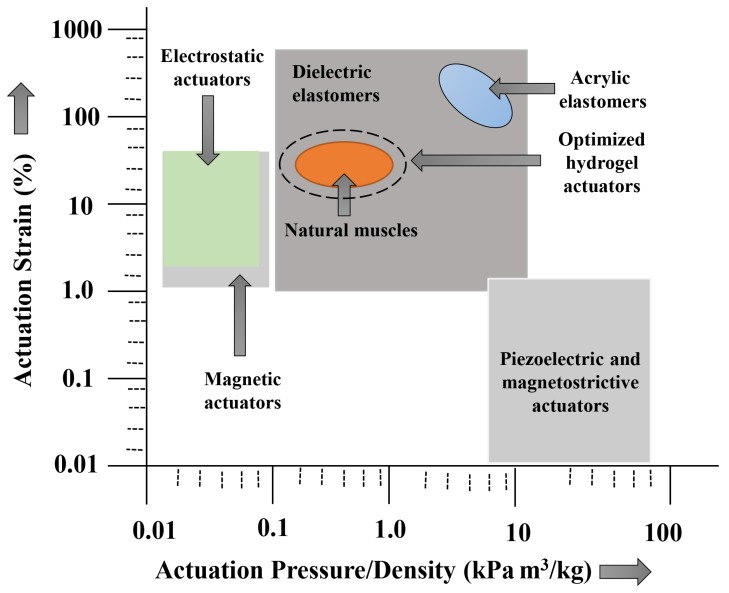
Different regimes of soft actuators and natural muscle. Optimized hydrogel actuators closely mimic artificial muscles’ tissue profile.

The design and implementation of hydrogel actuators in certain biomedical applications is currently under research and is very much in the developmental stage. For example, the application as artificial muscles [54] is not yet fully developed because of the lack of a strong material/gel, which is needed as a muscle carrying the load, and it also needs a combination of many smaller actuators [55,56,57,58]. Therefore, the current presence in the medical and engineering fields is embryonic. In Figure 1, we show different regimes of the actuation principle on the basis of the strain energy density function, for which the design goal was to make the hydrogel domain as close to natural muscles as possible, to biomimic in a better fashion. In the following sections, we cover different hydrogel-based actuators and their applications on the basis of the nature of the stimuli in biomedical fields. More precisely, we include here the most extensively studied hydrogel-based soft actuators, such as thermal, electric, magnetic, and biomimetic actuators; the relatively less-populated research conducted into hydrogel actuators being light- and pressure-driven, among others, is covered in a succinct way thereafter.

### 2.1. Thermo-Sensitive Hydrogel Actuators

**Figure 2:**
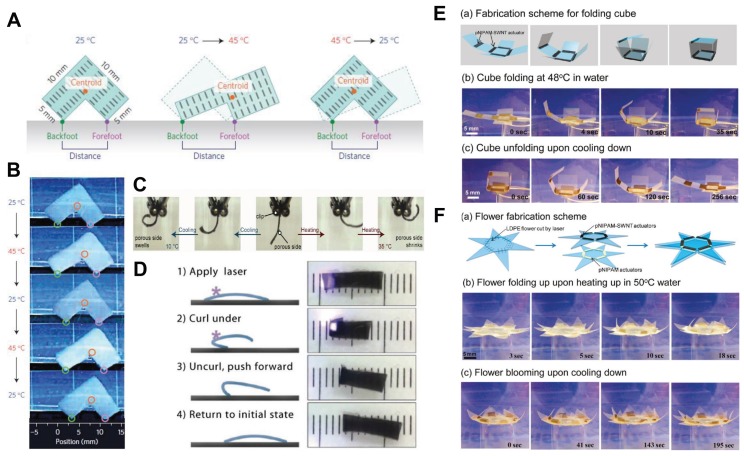
Thermo-responsive hydrogel actuators (HAs). (**A**) Basic diagram and mechanism for a unidirectional procession of L-shaped symmetric poly(*N*-isopropylacrylamide) (pNIPAM)/titanate(IV) nanosheet HAs (5 mm thick). (**B**) In the absence of substantial water uptake and release, distance between the nanosheets rapidly expands and contracts on heating and cooling, respectively, even in air, for which a hydrogel walker achieved a good forward motion. (**C**) Images of elastin-like polypeptide (ELP)–reduced graphene oxide (rGO) HAs reversibly curling in response to cooling or heating of the surrounding solution. (**D**) Photothermally actuated hygromorphic crawler. A hydrogel molded with a slight curvature was placed with porous side facing down. The laser was applied so as to induce gel curling. Subsequent uncurling during recovery after the laser was removed pushed the gel forward (1 mm tick marks). (**E**) Programmable cubes: folding cubes based on thermo-responsive HAs. (**a**) Fabrication scheme for folding cubes based on single-walled carbon nanotube (SWNT)–pNIPAM/low-density polyethylene (LDPE) bilayer actuators. (**b**) Cube folding by thermal actuation in 48 °C water. (**c**) Cube reversibly unfolded by cooling down the water bath in which the cube was immersed. (**F**) Programmable flower made by heterogeneous integration of pNIPAM and SWNT–pNIPAM bilayer actuators. (**a**) Fabrication scheme for making a programmable flower, consisting of two layers of actuators. (**b**) Flower folded (i.e., closed) when heated in a water bath to 50 °C. (**c**) Flower bloomed by cooling down in the water bath. (**A**,**B**) Adapted with permission from [79]. Copyright 2015, Macmillan Publishers Ltd. (**C**,**D**) Reprinted (adapted) with permission from [80]. Copyright 2013, American Chemical Society. (**E**,**F**) Reprinted (adapted) with permission from [64]. Copyright 2011, American Chemical Society.

Hydrogels have the property to change their volume and shape when heated above a particular temperature called the lower critical solution temperature (LCST), that is, the temperature below which the components of a mixture are miscible for all compositions. This shape/volume-changing property is useful for building actuators in soft robot and drug delivery systems. For example, thermo-responsive hydrogels, when combined with polymers, act as good drug delivery systems [59,60,61,62]. For example, a thermo-sensitive hydrogel, poly(*N*-isopropylacrylamide) (pNIPAM), was used to design a clamping mechanism in an inchworm-like robot by creating a difference in frictional force as a result of the hydrophilic (soft) and hydrophobic (tough) nature when heated below/above the LCST [63]. Additionally, pNIPAM, when combined with single-walled carbon nanotubes (SWNTs), can enhance the thermal response time of the actuation five-fold when compared to pure pNIPAM hydrogels, leading to more tunable and thermally responsive actuator systems. Water loss in the SWNT–pNIPAM layer results in a mechanical strain that causes the actuation of the bilayer at predefined sites [64]. Aside from pNIPAM, there are other thermo-sensitive hydrogels that have also been studied, such as poly(vinyl methyl ether) (PVME). Using water-swollen PVME and PVME fibrous gel, thermally activated chemo-mechanical devices such as an artificial muscle and finger, an automatic separation system, and a photosensitive device were modeled and evaluated [65,66,67]. Aside from this, there are hydrogels created by photopolymerization for cross-linking that also find uses in drug delivery applications and as cell encapsulation materials [17,68,69]. In Figure 2, we show some relevant examples of thermo-responsive hydrogel actuators that enhance the scope for further research in the biomedical field.

The thermally stimulated expansion and contraction of hydrogels are also used to maintain the flow of liquids in microfluidic systems and in the design of valves [70]. Further, hydrogels do not have any moving parts and can be easily combined with other substrates. Therefore, thermal hydrogels find applications in microvalve actuators and microfluids [71,72]. Using hydrogels for designing valves not only requires simple fabrication and operation, but also provides the ability to withstand high pressures and gives a perfect sealing. Additionally, the need of a sensor can be eliminated by extending to operate in a self-actuated, open-loop control mode. For example, a flexible microvalve diaphragm actuator was designed and fabricated using pNIPAM as a thermally responsive hydrogel actuated by flexible conductive nanocomposite polymer (C-NCP) heaters [70,73]. Hydrogel valves also find applications in flow control, sample and reagent metering, sample distribution into multiple analysis paths, and the sealing of polymerase chain reaction (PCR) reactors to suppress bubble formation [74,75]. Further, a microfluidic hydrogel actuator that uses a polydimethylsiloxane (PDMS) membrane to separate the hydrogel actuator from the channel and a separate reservoir for fluid to swell the actuator was designed and can control flow for organic and aqueous solutions with varying pH and ionic strength [76,77].

However, as described earlier, there are some limitations in using hydrogels here; pNIPAM gels require the presence of aqueous solutions with low ionic strength, which limits the types of fluids that can be used [78]. Hydrogels can swell with the fluid to block the channel, but this results in a constant total volume of the fluid and the gel, and hence the valve is operated with high polymer–solvent friction. Hydrogels inside a fluidic channel cannot be used to displace fluid [78].

### 2.2. Electro-Responsive Hydrogel Actuators

**Figure 3:**
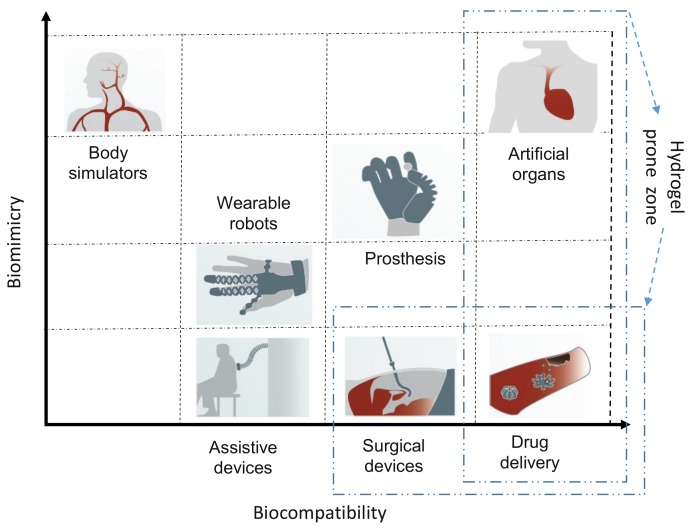
Biomedical soft robotic application areas from a materials’ perspective. The denoted region is best suited for hydrogel soft robotic applications in biology and medicine. Concept adapted with permission from [95]. Copyright 2018, Springer Nature Ltd.

Electro-responsive hydrogel actuators convert electrical energy given to them from the power supply and transfer this to mechanical energy through their active matrix deformations. One of the biggest advantages of using these actuators is their compatibility with electronics and batteries, making it easier to supply a power source. They generally find applications in artificial muscles, biosensors, and drug release systems (Figure 3). Neural prostheses (NPs), which help in supporting the damaged central nervous system, are also made using electro-responsive hydrogels [81,82,83]. Further, the shape-changing property of hydrogels and their bending under electric fields are the advantages of these composites and can be useful in the design of various actuators. Hydrogels such as poly(2-hydroxyethyl methacrylate) (pHEMA), hydroxyethyl methacrylate (HEMA), and polypyrrole are biocompatible, useful in designing biomedical devices, and also find uses in biosensors [82,84,85,86,87].

Like thermo-responsive hydrogels, electro-responsive hydrogels also posses a basic working mechanism. Their hydrogel actuators are generally made of composite materials—a combination of a hydrogel polymer along with conducting polymers (CPs) that are prepared from polyelectrolytes. Then, when an electric field is applied, the actuator swells/deswells depending upon the material and the experimental setup. Consequently, actuators made of these composites derive both sets of properties (electric conductivity from the CPs and the rigidness and the desired mechanical properties from the hydrogels). For example, CPs generally have inherently poor mechanical properties, but they can be polymerized to hydrogels with preformed hydrogel matrices and can be used as electrode coatings [88,89,90]. Additionally, there are double-network (DN) hydrogels, which improve the elasticity of the material [91], and oriented graphene hydrogels (OGHs) are conductive gels [92,93] that are elastic and deformable, can be designed into thin films, and are used in biomedical devices for which rigidity is not the concern [94].

Drug delivery systems are the main area in which electro-driven actuators are used because of their simple functioning logic—the drug is released either through diffusion or shrinking of the gels when the electric field is supplied; they are therefore also called electro-simulated drug release devices (EDRDs) [96,97]. In one case, a polyelectrolyte hydrogel was kept between a pair of electrodes that deswelled under an applied direct current (DC) voltage, releasing water, and hence could be used in electrically stimulated drug release devices, deep brain stimulation electrodes, and low-voltage actuation [98,99]. However, using electro-responsive hydrogels leads to a slow response time, and high stress is induced in the gel, leading to fatigue. To counter the slow response time, either the hydrogel units can be reduced in size or sol–gels (called smart glasses), which have a fast response time, can be used. Another disadvantage is that the drug release versus current is proportional only for small gel contractions.

**Figure 4:**
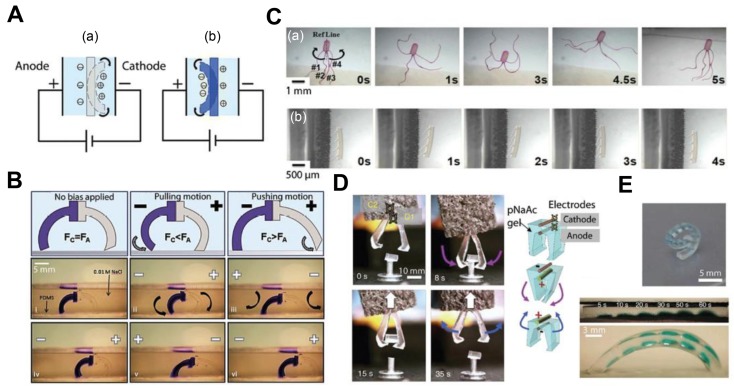
Ionic-hydrogel-based actuators. (**A**) Basic diagram of mechanism with (**a**) anionic and (**b**) cationic gels in the same solutions; each could achieve opposite bending directions under the same applied field. (**B**) Walker achieved unidirectional motion using both anionic and cationic gels. A field of 5 V/cm was applied; electrode field directions are shown in the images. (**C**) Locomotion of an anionic gel (**a**) octopus and (**b**) walker under electric fields in solution; the applied voltage signals were alternating (+7 V/−15 V) and ±15 V, respectively. (**D**,**E**) Patterning of hydrogel using ionoprinting for directional embedding of ions. (**D**) A gel gripper was able to grasp and release items. (**E**) The imprinted area size and location affected the degree of bending. (**A**,**B**) Adapted with permission from [52]. Copyright 2014, The Royal Society of Chemistry. (**C**) Reproduced with permission from [105]. Copyright 2008, John Wiley & Sons Inc. (**D**,**E**) Adapted with permission from [106]. Copyright 2013, Macmillan Publishers Ltd. The entire figure adapted with permission from [107]. Copyright 2017, John Wiley & Sons Inc.

On the other hand, tests and experiments are being conducted to explore the usage of these materials in emerging fields such as artificial muscles and walker robots. A study was conducted with poly(acrylic acid) (PAAc)/poly(vinyl alcohol) (PVA) copolymer hydrogels to use them as base materials for building such applications [100,101]. Here, the use of a composite is encouraged, as for every actuator, to obtain the desirable features of different materials. An ionic polymer gel—a composite of PVA and poly(sodium acrylate)—was designed and demonstrated an electric field-associated bending motion in aqueous solutions, which was developed into an electrically driven polymer gel finger having muscle-like properties and good sensitivity to electric signals [102]. This was extended to SWNTs/PVA and multi-walled carbon nanotubes (MWNTs)/PVA, which showcased better actuating properties [103,104]. Another test was conducted by designing a dual-legged walker by binding the legs with two types of polyelectrolyte hydrogels that bend in opposite directions with respect to an electric field, which engages motion on surfaces [52]. Electro-responsive hydrogels can be used in various places because of their electric compatibility, and their disadvantages can also be compensated for by bonding to new materials. For example, degradable hydrogels are being designed, as the previous electro-responsive hydrogels were not degradable, which is a major drawback for use in the biomedical field [10,81]. There has also been a study of an actuator that involved a combination of thermo-responsive and electrostatically anisotropic hydrogels for permittivity switching [79]. In Figure 4, we show some relevant examples of micro-/nanoscale soft robots based on ionic hydrogel actuators with an immense potential for biological and biomedical applications.

### 2.3. Magneto-Responsive Hydrogel Actuators

Magneto-driven actuators are actuators that produce actuation and motion driven through magnetic fields [108,109]. To obtain an idea about the workings of magnetic actuators, there is a study on a hydrogel-based microgripper that was designed and can be moved freely in liquids when DC magnetic fields are applied and that performs gripping when alternating current (AC) fields are applied [110]. These actuators are becoming a demanding product in biomedical applications such as drug delivery and cell design. To name a few, anisotropic or isotropic shape changes can be made in hydrogel-based composites by application of an external magnetic field that depends on the magnetic particle content and the particle saturation magnetization; these find uses in medical implants, heart pump components, or micropumps for controlled drug delivery [111,112]. In another example, ocular drug delivery systems were designed for microrobots with magneto-actuated hydrogels that focus on targeting drug delivery in the eye [109,113].

The main principle behind actuation is that magnetic particles that are embedded in the actuators act in response to the external magnetic forces applied, causing the system to bend, expand, or deform in other ways [114,115]. Further, we have two ways to make magnetic soft actuators—either completely cover the actuator with magnetized particles or add discrete magnets to them at different places. The former method is complex and expensive, but it can lead to excellent actuation of the hydrogels. The magnetic particles can even be microfluids such as magneto-rheological fluids (MRFs) [116]. In one study, microfluidic-based synthesis of spherical magnetic hydrogel particles was used for the microparticles to exhibit superparamagnetic behavior, which is highly demanded in biomedical applications [117,118,119]. The direction and the magnitude of the magnetic field can be varied, leading to different motions of the bodies.

As we can say generally that hydrogels are biocompatible and can bond with other polymers through processes such as cross-linking, magneto-responsive hydrogels can also become more useful, particularly in controlled drug release through magnetic actuation. For example, advancements were made by creating a hybrid magnetic carboxymethylcellulose (CMC) hydrogel with CoFe${}_{2}$O${}_{4}$ nanoparticles acting as cross-linkers aiming for controlled drug release [120,121]. Further, nanocomposites of hydrogels, particularly magneto-responsive hydrogels, have been combined with MWNTs and find uses in applications such as tissue engineering [19] and remote-controlled drug release devices [122,123].

**Figure 5:**
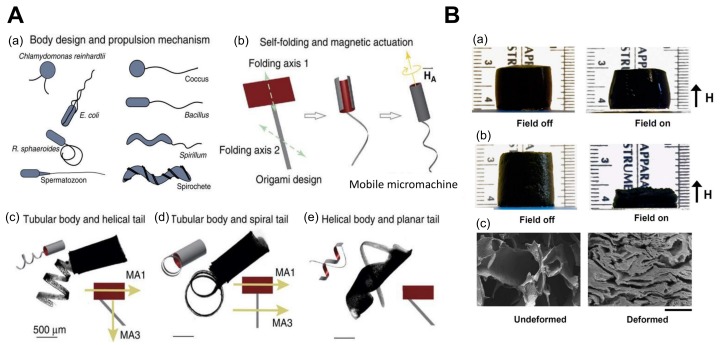
Magneto-responsive hydrogel-based actuators. (**A**) Reconfigurable body plans for soft hydrogel-based micromachines inspired by microorganisms. (**a**) A variety of hydrogel-based body designs and propeller mechanisms. (**b**) Anisotropic swelling behavior controlled by the alignment of magnetic nanoparticles (MNPs) along prescribed three-dimensional (3D) pathways and selective patterning of supporting layers results in 3D functional micromachines. The folding axes 1 and 2 denote the direction of folding for each compartment. The micromachine possesses multiple different magnetic axes, which determine the motility when the magnetic field (MF) is applied. The flagellated micromachine, which contains self-assembled MNPs, performs controllable swimming in the 3D space under a homogeneous rotating MF. (**c**–**e**) Optical images of flagellated soft micromachines with complex body plans. Magnetic axis (MA1 and MA3) denote the magnetic axes in the head and tail, respectively. Scale bars: 500 μm. (**B**) The active hydrogel scaffold undergoes a large deformation and volume change via a moderate MF. (**a**) A cylinder of nanoporous hydrogel was reduced by nearly 5% of its height when subjected to a vertical MF gradient of 38 Am${}^{-2}$. (**b**) The corresponding macroporous hydrogel deformed by nearly 70% under the same MF. (**c**) Scanning electron microscopy (SEM) images of a free-dried macroporous hydrogel in the undeformed and deformed states. Scale bar: 500 μm. (**A**) Adapted with permission from [124]. Copyright 2016, Springer Nature Ltd. (**B**) Reproduced with permission from [125]. Copyright 2011, National Academy of Sciences.

There are a few reasons that magnetic hydrogel actuators might in fact be the best actuators used for biomedical applications in the near future. A relatively higher actuation force is released upon generating the magnetic field. Additionally, they can be used in most mediums, as magnetism has a high penetrability in various materials. Another benefit is that they have a faster response rate than their counterpart actuators. This suggests that they can be an important alternative for thermal and electric stimuli-based actuators in terms of better control of actuation and a large effective output. However, one disadvantage is that they have a high power consumption for larger workspaces, and hence, there have not been many experiments or implementations using magnetism in hydrogel actuators to date. In Figure 5, we show basic principles of design and development of mangeto responsive hydrogel-based actuating systems.

### 2.4. Biomimetic Hydrogel Actuators

**Figure 6:**
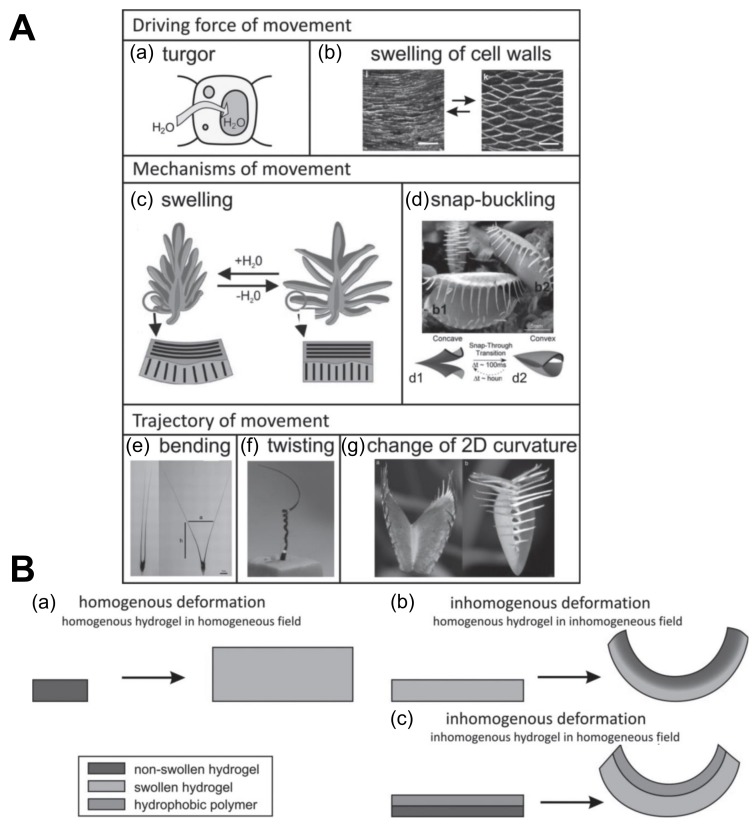
(**A**) Examples of reversible movement in plants with respect to the driving force: (**a**) turgor, and (**b**) swelling of cell walls; mechanism: (**c**) swelling, and (**d**) snap-buckling; and trajectory: (**e**) bending, (**f**) twisting, and (**g**) change of two-dimensional (2D) curvature. (**B**) Different scenarios of swelling of hydrogels: (**a**) homogeneous deformation of homogeneous hydrogel; (**b**) inhomogeneous deformation of homogeneous hydrogel; (**c**) inhomogeneous deformation of inhomogeneous hydrogel. (**A**,**B**) Adapted with permission from [8]. Copyright 2013, John Wiley & Sons Inc.

Living organisms have undergone evolution and have adapted to various environmental responses and factors over millions of years. Many inventions have resulted from close observation of nature’s creations and lifestyles and by mimicking the solutions for their day-to-day problems. For example, the wide range of morphologic and functional mechanisms in plants with swelling/deswelling at the cellular level making plants lean towards sunlight while growing [126,127,128]. In the plant kingdom, this swelling/deswelling process can lead to bending, expanding, contracting, twisting, fast buckling, and even fracture [129]. In the same way, synthetic hydrogels can also mimic the behavior of plant cells [10,130,131]. The term we use for mimicking living models to solve complex human problems and to make advancements is biomimetics. Biomimetics has given rise to new technologies inspired by biological solutions at the macro- and nanoscales. As an example, artificial bone marrow was designed using synthetic polyethylene glycol (PEG) hydrogels [132,133]. Additionally, such artificial models can be used for soft robotics simulation and control with software such as SOFA (http://mimesis.inria.fr/), and external forces can be applied to them to see how they respond to. In Figure 6, we show some related biomimetic principles and actuators inspired by nature for soft robotic application.

Hydrogels can play a crucial role in biomimetics as a result of their stimuli-responsive capabilities and biocompatibility. Adding to the benefits, their functional and mechanical properties can be improved by forming composites with materials having toughness and bendability. For example, chitosan hydrogels were used to mimic tissues and cells and can also be enhanced to protect from wear and tear through structuring [134,135,136,137]. Equally, biomimetic actuators were inspired and developed from the movements, features, and functions of plants. Plants, although being apparently stationary, undergo subtle movements such as tropisms and nastic and tactic movements, such as in the case of the plant *Mimosa pudica*, which is a sensitive plant that folds inwards when touched. Inspired by *Mimosa*, researchers have developed a reversible temperature-responsive bilayer hydrogel with an internal water circulation that can work under different phases [8,138]. In other studies, the use of the stratified deformation of inhomogeneous hydrogels helped in mimicking the reversible movement of plants such as pine vines and Venus flytraps (*Dionaea muscipula*) [8,139]. Similarly, the same principle can be extended to insects and fishes with unique features, and there are examples to support this. Inspired by the *Morpho* butterfly, biohybrid structural color gels can be useful in designing multi-colored living materials [140,141]. Other researchers aimed to optically and sonically camouflage hydrogel actuators in water by trying to achieve a high optical and sonic transparency in water by mimicking the leptocephalus natural camouflage (hydrogel robotic fish) [6].

#### Key Limitations

However, there are various challenges that strongly limit the use of hydrogels in biomimetics. Because of their inherent mechanical weakness, they cannot withstand high load-bearing stress. Additionally, hydrogels cannot be sterilized easily because of their high water content, and sterility is a core aspect in the biomedical field. Despite this, there are ways of designing composites to overcome the shortcomings of hydrogels. Hence, we envision that hydrogels will likely be an important material in biomimetic actuating systems and models in reality soon. In fact, hydrogel tissue scaffolds have already become predominant in tissue engineering applications and are replacing those made using plastics. In Table 3, we elucidate some pros and cons of using different stimuli in hydrogel actuators and relevant literature for further reference.

### 2.5. Miscellaneous

Smart variable stiffness actuators using polymer hydrogels were designed to work on controlled gel swelling in the presence of water. They restrict the free swelling of the gel and reduce the gel expansion in the radial direction, maximizing expansion/contraction in the axial direction [110,150,151]. In one study, an omnidirectional multi-material cylindrical actuator using many polymers was designed for applications in which controlled, complex, precise, and fast motion is required [152,153,154,155].

## 3. Hydrogel-Based Biomedical Sensors

**Figure 7:**
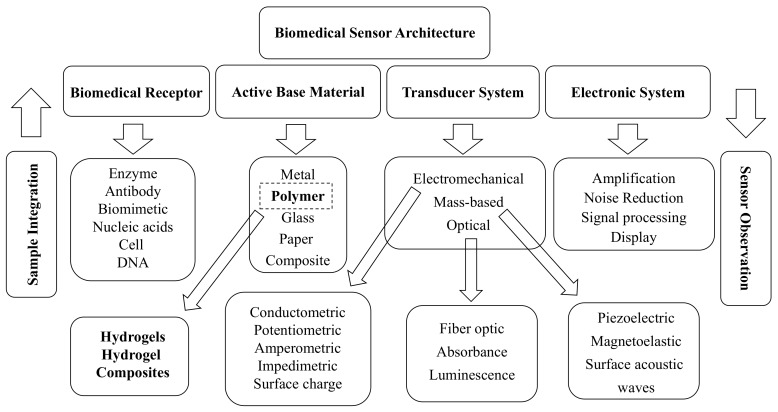
Overall schematic of a hydrogel-based sensor architecture. Concept adapted with permission from [162].

Biomedical sensors are transducers that convert external forces/stimuli into bioelectromechanical signals to feed to the input. These external forces may be of any type—chemical, optical, magnetic, mechanical, acoustic, or biological [156,157]. For example, the color change feature of photonic crystal (PC) hydrogels as a result of swelling on contact with water helps them to act as humidity sensors [158]. Similarly, in gas sensors, hydrogels are mainly used as a protective coating for the sensor and for sensing different gases through generating forces or swelling when exposed to certain types of gas [156,159,160]. These sensors have proven themselves to be a great aid, particularly in the medical field for various processes and operations such as surgeries/treatments that require the constant monitoring and measuring of external and internal dynamic variables (e.g., blood pressure) with an output easily understandable by the user. We find the wide usage of hydrogels as active components to fabricate better miniaturized biomedical sensors as a result of their optimized swelling/shrinkage properties and customizable features (e.g., changing densities, mass, volume, and stiffness) [161]. In Figure 7, we show the biomedical sensor architecture and the paradigm in which hydrogel sensors can play an impotant role.

Stimuli-responsive hydrogels are used as sensing materials, as they reflect small changes in particular environmental variables with their volume/mechanical properties. However, we need to consider the precise selection and fabrication of these materials, as the response speed depends on the hydrogel’s composition, shape, and size. Aside from sensing, hydrogels can be used in sensors as a defensive cover or coating for the protection of sensor parts from interactions with unnecessary molecules or cells, and further, they can also be used as stabilizers. Like actuators, there are hydrogel sensors that use different stimuli and mechanisms for detection of the external signal. They are also used as an immobilization matrix for sensors [163,164]. In the biomedical field, the main function of hydrogels for biosensors is for the protection and coating of sensor parts to prevent reactions with external factors in the tissues or cells [165]. There are various factors to be measured and sensed in biomedical processes, treatments, research, and so forth [166,167,168,169], which are briefly discussed in the following sections.

### 3.1. Glucose Sensors

Glucose levels in the blood play a pivotal role in determining the blood sugar amount secreted in the body. As millions of cases of hyperglycemia (high sugar) and hypoglycemia (low sugar) occur suddenly, and as we do not have a permanent cure for these, frequent supervision of sugar levels is necessary and has been further extended to constant glucose monitoring (CGM), in which the glucose level is monitored all day and all night.

In this realm of glucose monitoring and for patients suffering from hyperglycemia/hypoglycemia, hydrogel sensors can play a vital role to detect the body’s glucose level mainly by the addition of boronic acid, a glucose-sensitive monomer. Glucose molecules diffuse into the sensor and bind reversibly with boronic acid groups in the hydrogel via affinity binding, causing changes in the dielectric properties of the hydrogel, which can be measured using a microelectromechanical system (MEMS) capacitive transducer to determine the glucose concentration [170]. Cyclic boric and boronic esters are formed when boronic acids react with saccharides such as glucose, leading to a pH change, which is measured as the output [171]. To learn more about the chemical structure and properties of the hydrogel–boronic acid composition, phenyl boronic acid (PBA) was added to hydrogels and studied via experiments [172].

In recent years, there have been various modifications and improvements to these sensors. In one study, the performance of the boronic glucose sensor was improved by linking the hydrogel to a quartz crystal microbalance (QCM), resulting in real-time monitoring [173]. In another case, boronic acid was added to silver nanoparticles embedded in silica-coated graphene oxide (GO) via surface-enhanced Raman spectroscopy (SERS) with a range of 2–20 mM glucose detection [174]. Additionally, in another attempt, the glucose sensor was made wireless using an ultra-low power tunnel oscillator [175]. Furthermore, a volume resetting agent was added to the sensor to obtain a linear and fast response [176]. In addition to pH variation, another method was also developed on the basis of fluorescent sensing—the first fluorescent glucose sensor was designed by Yoon and Czarnik [177]. By analyzing the spectroscopic properties of boronic acids, it was found that certain signals can be generated when the monomer is bonded with diols and saccharides. Different types of this fluorescent signal were studied for boronic compounds to implement them in glucose sensors [178]. The fluorescence change upon binding is believed to be caused by changes in the electronic properties accompanying rehybridisation in boron from sp${}^{2}$ to sp${}^{3}$ [171]. In Table 4, we show different chemicals used for hydrogel-based glucose sensing and their key performances with references to relevant literature.

### 3.2. Touch/Stress/Stretch Sensors

**Figure 8:**
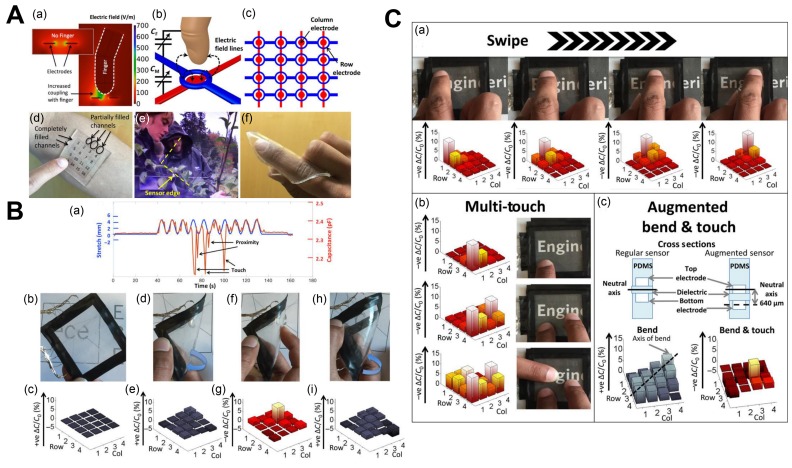
A touch sensor using stretchable and ionically conductive hydrogel electrodes, which project electric field above the sensor to couple with and sense a finger. (**A**) Working principle and properties of the touch sensor; (**B**) touching the sensor while sensing and bending; and (**C**) multi-touch, swipe, and augmented bend detection. Reproduced with permission from [190]. Copyright 2017, AAAS.

Touch/tactile sensors are one of the most basic sensors and convert bending/deflection to force, stress, or strain. Robotic surgeries such as minimally invasive surgery (MIS) and incisions are becoming common as a result of their accuracy and the painless background generated. However, surgeons become devoid of the feeling of touch during the process, which is required for safety measures of the autonomous procedure. Therefore, biomedical touch/pressure sensors are required for these applications [189]. Because all hydrogels have swelling and volume-change properties, a variety of touch/stress sensing applications and devices can be designed and studied using various additions to the sensors, particularly with electroactive hydrogels [190]. The swelling pressures of hydrogels for various stimuli were measured using piezoresistive pressure sensors with perforated diaphragms [191]. For example, a touch sensor made of stretchable and ionically conductive hydrogel electrodes was designed to create an electric field to couple with and sense a finger during bending [190,192,193,194,195]. In another study, conductive hydrogels were studied for touch-sensitive and stress-locating features for soft robotics [192]. In Figure 8, we show some examples of biomedical tactile sensors and their function.

The mechanical deformations of hydrogels under external pressure pave the way for them to act as a sensor. These sensors find applications in measuring the pressures of blood and oxygen and are used in smart skin and touch-sensing applications, as well as in other equipment during treatment. Additionally, there is another study on strain sensing hydrogel optical fibres in which dye-doped hydrogel fibres were used for measuring axial strain and distributed strain sensing with a large dynamic strain range. Here, a hybrid of ionic and covalent polymer networks was formed whereby the covalently cross-linked polymers gave high stretchability and the ionically cross-linked polymers increased the hydrogel’s strength by releasing mechanical energy during bending [190].

Furthermore, they can be copolymerized with stronger polymers such as SWNTs to enhance strength with sensing capabilities. In one study, a highly sensitive, low-cost, wearable pressure sensor was designed using SWNTs combined with conductive hydrogel spheres, which are useful in health monitoring [196]. Conductive hydrogels have also been studied for touch-sensitive and stress-locating features for soft robotics. These hydrogels can be used for detecting stretching and contact by combining the soft-wet feature of the hydrogels with the electrical properties given by the dissolved ions [197]. Additionally, it has been attempted to make elastic conductive hydrogel sponges by conversion to a stress sensor [198].

### 3.3. Ionic Strength Sensing for pH Ions

The pH measurement is extremely important in many areas of biomedical applications, as slight changes can cause unnecessary reactions that may harm the devices or the patients. A continuous pH value needs to be measured in blood, as well as in aqueous solutions of different materials such as glucose, medicines, and more. There are various pH sensors, starting with the basic potentiometric sensor consisting of two electrodes, including a reference electrode. Here, we focus on pH measuring devices using the material properties of hydrogels. Sensors that are pH sensitive and hydrogel-based are used to measure pH with high accuracy, as their swelling/shrinking properties have a close relation with pH value for solvent concentrations of aqueous solutions. The swelling/shrinking results in a change in volume, which can be transduced to the required electric force. A common conversion of these signals is by using silicon-based piezoresistors. In one of research work, a hydrogel was composited with a piezoresistive sensor with silicon as the base material, which, upon transferring the deflection from the hydrogel, resulted in electric signals. Then, the output voltage versus pH characteristics were studied [199]. Another study was also done on a hydrogel-based piezoresistive pH sensor, whereby the deflections were simulated using Ansys [200]. The working range of sensors makes it less difficult to measure pH with a single equivalent sensor.

Additionally, there are alternatives to piezoresistive sensors. Polyelectrolyte hydrogels are composed of ionisable weak acidic and basic groups. A phase transition of the hydrogels takes place in a small range near to the acid dissociation constant (pKa) of the hydrogel. The ionisable group of the gel is the main factor for pH sensitivity [161]. An example for the use of pH sensitive hydrogels based on an intelligent polymerized crystalline colloidal array (IPCCA) was developed by Reese et al. [201]. Further, there are pH responsive hydrogels used in actuations as well as in drug delivery. In one case, these hydrogels were magnetically actuated in a soft microrobot and were used for drug targeting and delivery. The soft microrobot with eight radial arms had a bilayer structure of biocompatible pHEMA and poly(ethylene glycol) diacrylate (PEGDA) with Fe${}_{3}$O${}_{4}$ nanoparticles [202]. Additionally, hydrogels that are responsive to pH were used for dual drug delivery systems, adding the capability to be made biodegradable [203].

### 3.4. Temperature Sensors

Temperature plays a key role in biological reactions as well as in the functioning of biological machines. Taking the simple case of a hospital, the temperature of blood, respiratory air, incubators, cooling systems, and many more need to be measured. Therefore, temperature sensors are expected to be designed and built in such a way so as to have the maximum precision and accuracy. The usage of thermo-responsive hydrogels in temperature sensors is widely followed because their shape/volume changes with respect to the temperature, from which the exact value of the temperature can be calculated. The commonly used thermo-responsive hydrogels are pNIPAM and other cross-linkable polymers such as *N*,*N*-dimethylacrylamide (DMAAm) and 2-(dimethyl maleimido)-*N*-ethyl-acrylamide (DMIAAm). A wide variety of swelling behaviors with respect to the temperature change, different needs and ranges of temperature measurements in the biomedical field can be found out. Additionally, some methods have been suggested to improve the properties of the thermo-responsive hydrogels such as pNIPAM in chemical sensors [204]. The other methods for temperature sensing are an extension of the above—using fluorescence and luminescence, which are generated as a result of spectral behaviors of these hydrogels at different wavelengths. For example, a temperature sensor that senses through spectral shifts in quantum dots was designed [205]. In another study, dipyrazolyltriazine tris(β-diketonate) europium(III) complexes were used as luminescence temperature sensors that were combined with a polyurethane hydrogel matrix to provide sufficient stability of the probes [206]. Additionally, there has been interesting research done on the dual luminescence of hydrogel nanoparticles responsive to both temperature and pH that also produce emissions strongly and independently. The red emission pertained to a temperature change ranging from 10 to 80 °C, while the blue emission showed a linear response between pH values of 6.5 and 7.6 [207]. In one study, IPCCA changes in volume with respect to temperature caused a colo transformation due to diffraction [156]. There have been various studies on temperature sensors. For example, hydrogel PCs are materials that swell as a result of temperature changes, and the lattice distance thus increases, changing the photonic stop band proportionally to the temperature change. These hydrogels are also used to act as stabilizers in these sensors [208].

### 3.5. Living Hydrogel Sensors

**Figure 9:**
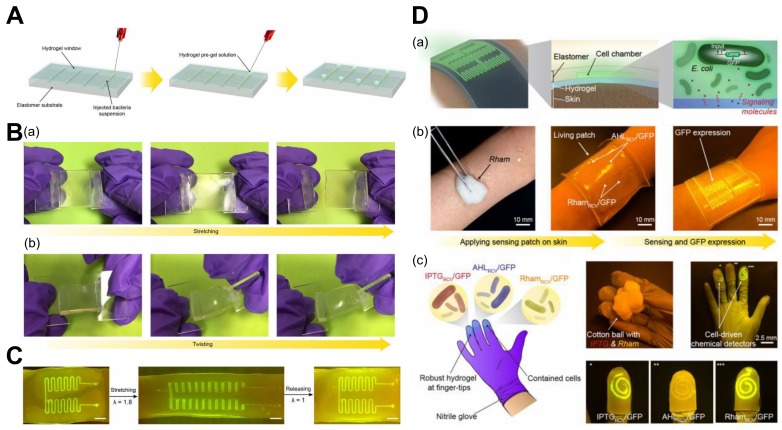
Living materials’ design and devices based on stretchable, robust, and biocompatible hydrogel–elastomer hybrids that host various types of genetically engineered bacterial cells. (**A**) Schematic illustration of cell suspension injection and sealing of injection points; (**B**) deformation of agar-based living devices; (**C**) functional living device under large uniaxial stretch; and (**D**) living hydrogel-based wearable devices. Reproduced with permission from [209]. Copyright 2017, National Academy of Sciences.

There are other ways hydrogels can be exploited in sensing aside from bending and shape changing. These are generally by direct interaction with molecules, antigens, and antibodies. This also results in biochemical sensing mechanisms for hydrogel sensors. Furthermore, composites of hydrogels combined with living cells and organisms are formed for biosensing applications and are called living sensors. This synthetic biology can harness great results in deformable cells with living capabilities. In one such study, hydrogel–elastomer matrices were combined with programmed living cells, resulting in stretchable living materials used as living sensors, genetic circuits, and wearable devices [209] (Figure 9).

Even in these living and cell sensors, a hydrogel coating can be used to prevent deposits from entering the sensor. Additionally, there has been a development in lanthanide luminescence-based sensing of enzymes in the body with the help of hydrogels, as this is preferred over fluorescence-based sensing because of interference. Recently, there was a breakthrough at the Massachusetts Institute of Technology (MIT), where wearable living sensors with programmed cells in the form of gloves and bandages were designed that could detect certain chemicals by glowing/lighting up.

### 3.6. Biochemical Sensing Mechanisms

There are other means of sensing for hydrogels aside from bending and shape morphing, such as to calibrate the biochemical environment for vast biomedical applications. Even in these living and cell sensors, a hydrogel coating can be used to prevent deposits from entering the sensor. In one study, the living sensor was made stretchable and robust by combining it with hydrogel–elastomer matrices [47]. Additionally, there has a development in lanthanide luminescence-based sensing of enzymes in the body with the help of hydrogels, as this is preferred over fluorescence-based sensing because of interference [166,185,210].

## 4. Different Hydrogel Structures: An Overview from a Mechanical Standpoint

**Figure 10:**
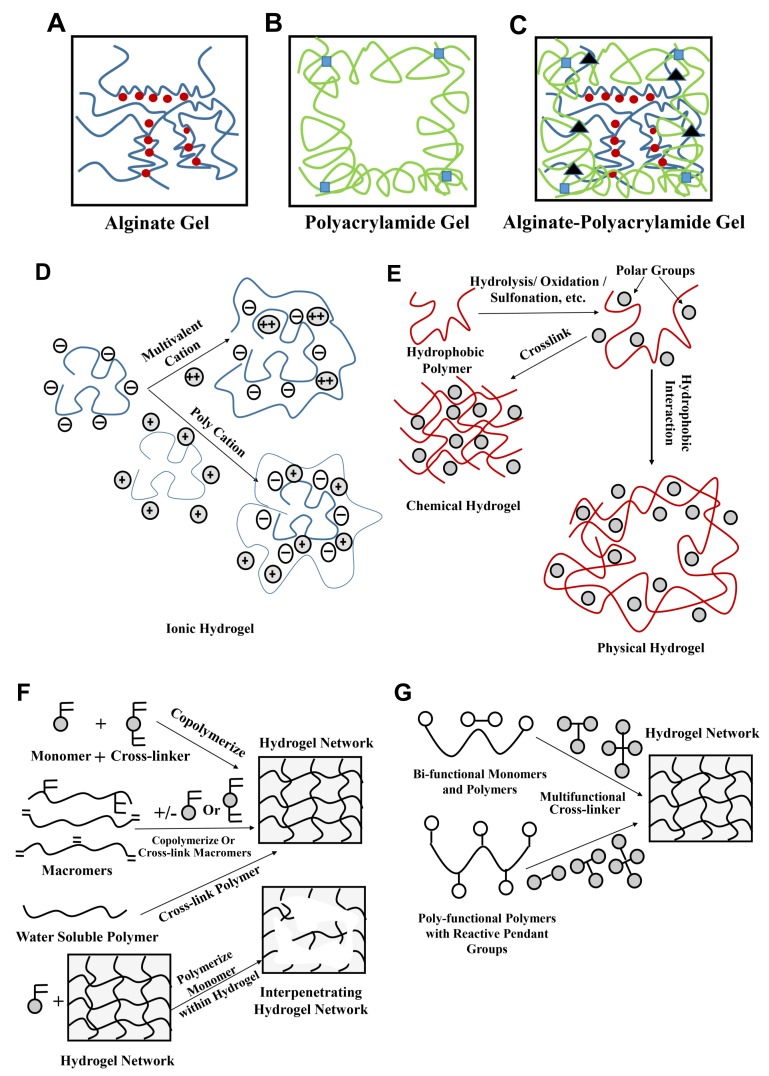
Schematic representation of different types of hydrogel polymer internal structures. (**A**) Representation of an alginate gel for which G blocks of ionic polymeric chains cross-link via Ca${}^{2+}$ ions (red circle). (**B**) Internal structure configuration of a polyacrylamide gel for which *N*,*N*${}^{\prime }$-methylene-bis-acrylamide (MBA) (blue square) acts as a covalent cross-linker. (**C**) An example of alginate–polyacrylamide hybrid gel, where both alginate and polyacrylamide networks are integrated through a strong covalent cross-linker (black triangle): carboxyl groups for alginate gel and amine groups for polyacrylamide gel. The components required for this biocompatible alginate–polyacrylamide hybrid gel are deionised (DI) water, acrylamide (AA), sodium alginate (SA); 2-hydroxy-4${}^{\prime }$-(2-hydroxyethoxy)-2-methylpropiophenone (photoinitiator); *N*, *N*${}^{\prime }$-methylene-bis-acrylamide (MBA), customized ultraviolet (UV) light exposure, and resting time. (**D**) Schematic representation of two types of ionic hydrogels. (**E**) Schematic representation of hydrogel formation with chemical modification process inherited for hydrophobic polymers. (**F**) Schematic of hydrogel preparation though cross-linker and free radical reaction. (**G**) Schematic representation for formation of hydrogel cross-linked polymer through condensation reaction of diverse reactants.

Since the inception of HEMA hydrogels in 1960, as a result of the incredible work by Wichterle and Lim [211], hydrogels have long been the centre of attraction for biomedical engineers and scientists [212,213,214,215,216,217,218,219]. Generally, hydrogels consist of a network of polymer chains that swell in aqueous conditions to release their pre-loaded contents. They consist of either physical or chemical cross-links with pockets of an aqueous medium, which allows them to swell. The term “cross-link” refers to the connection point of several polymeric chains (Figure 10). These junctions are usually small chemical bridges; however, they can also be the association of macromolecular chains caused by van der Waals forces or hydrogen bonds [220,221,222]. For a long time, different chemical components have been tested to act as reinforcements and form composites with hydrogel systems to generate more robust and biodegradable structures. Additionally, these are human tissue friendly and hence have been applied for in vivo cell encapsulations [223,224,225,226] and in tissue and organ regeneration [227,228]. Hydrogels have also been widely used in various drug delivery applications [10,229,230]. Many of these hydrogel polymer matrices are predominantly single-network hydrogels, meaning that they have a single cross-linked polymer network [3,10,231]. However, these hydrogels, despite their biocompatibility and biodegradability, lack the mechanical strength and elasticity for the fabrication of soft robotic actuators. Here, we touch upon a brief introduction of some familiar hydrogels and synthesis mechanisms (Figure 10), and later we illustrate the same in actuator and sensor applications, extrapolating mostly to biomedical-related applications, and present the challenges to be overcome in the near future.

### 4.1. Single-Phase Gels

Hydrogels can be classified as single- or multi-phase gels. Single-phase gels are those that do not contain external discrete particles, and the appearance of the gel is clear and homogenous. Furthermore, they are the most common gels found naturally; their composition is mostly water, and the polymer content constitutes less than 10%. However, regarding their applications in actuators and sensors, we find low polymer content gels to be weak and easily dehydrated—water-retaining capabilities are essential for various mechanisms. Therefore, gels with a higher polymer content are preferred for better rigidity and water-absorbing characteristics. Some common organisms and cells that consists of these types of gels include soft weeds, sea anemones, and human cartilage, corneas, dermises, and arterial walls. On the other hand, these structural gels are also present in synthetic materials such as plasticised polymers.

Although single-phase gels are commonly present, their mechanical properties have not been studied properly because of their weak nature and, at present, they are not being used widely for biomedical applications. In addition to this, they have nonlinear relationships for stress–strain, and completely different reactions take place for these soft materials when exposed to stresses compared to those of the usual rigid structures that are familiar to everyone. Thus, as reported by Calvert [4], there are two important mechanical properties to be considered while designing single-phase hydrogels: elastic moduli and the measurement of the gel strength. The elastic moduli of hydrogels are not 100% linear, as the material changes its behavior at elastic–plastic transitions and is dependent on factors such as temperature, water content, ionic content, and density. On the other hand, synthetic gels are not strong enough to be used in structural applications; they are not even stronger than the naturally occurring gels, and the reason for this is the structural arrangement of these gels. Furthermore, strength can be determined from the amount of force under which deformation and bending occurs. Therefore, for measuring the gel strength, trouser tear tests [232] and tensile impact tests are used. Alternatively, the strength can be measured using the Griffith equation [233], where the variables are the fracture surface energy, elastic modulus, and crack length.

### 4.2. Multi-Phase Gels

These gels are useful in overcoming the disadvantages of single-phase hydrogels and are extended to DN hydrogels. These hydrogels consist of different phases, are heterogenous, and are in general turbid. In these gels, cross-linking of different materials and fiber reinforcement are present, combining the properties of different materials. Additionally, they contain crystalline structures, which can also be altered for enhancement of their features. The main reason for the separate phases generated in these hydrogels is due to extensive cross-linking and therefore impediment of the entropy and growth of polymer chains, making the distinct phases clearly visible [20].

Multi-phase gels have some advantages, such as increased elastic moduli, strengths, or stretching. These are mainly because of the cross-linking of two different polymers with mutually beneficial features. Furthermore, there are reports of producing high water content gels by reinforcing or cross-linking with the required polymers. Poly(vinyl alcohol) gels can be enhanced by increasing their strengths and moduli by adding dimethyl sulfoxide (DMSO) as a cosolvent, by exposing to a series of freeze–thaw cycles, or by mixing different solutions via irradiation [234]. As we can increase the strengths and moduli of the hydrogels in the multi-phase design, we should be careful to maintain the stiffness of the gels, not to increase it.

### 4.3. Double-Network Gels

In many cases, hydrogel compounds behave hydrophobically and cannot retain fluids from the body for treating wounds. Previous methods for DN hydrogel synthesis include a multi-step free-radical polymerization process [4,235,236,237,238], where strong polyelectrolytes are used to form the first physical network, and neutral monomers, initiators, and cross-linkers are used to form the second network. However, Chen et al. [239] observed many limitations in this method, including the long fabrication process and difficulty in controlling the molar ratio of monomers (not reproducible). Therefore Chen et al. [239] introduced a new one-pot synthesis procedure. However, this method also has its drawbacks: (i) the customizability of mechanical properties by altering key variables is not explored; (ii) the efficiency of these hydrogels in delicate structures and in soft robotic applications is not explored. Therefore, the key variables of fabrication are, namely, (i) the amount of time for exposure to UV irradiation; (ii) the cross-linker concentration; and (iii) the amount of resting time, which is of great importance, while the ease of fabrication is maintained.

**Figure 11:**
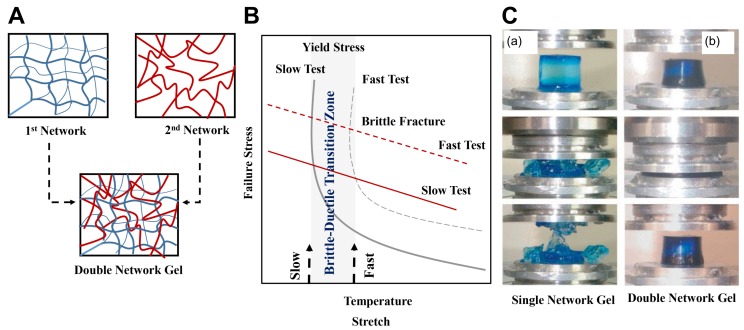
Hydrogel network formation and mechanical strength testing. (**A**) A typical example of double-network (DN) hydrogel formation. (**B**) Representation of the transition from brittle to ductile structures in a polymer network. Adapted with permission from [4]. Copyright 2009, John Wiley & Sons, Inc. (**C**) Compression of (**a**) single-network gel, and (**b**) DN gel illustrating how DN gel can sustain high compression. Reproduced with permission from [91]. Copyright 2003, John Wiley & Sons, Inc.

In this realm of strong DN hydrogel synthesis, Pacelli et al. [240] stated that the incorporation of gellan gum (GG) polyethylene glycol dimethacrylate (PEGDMA) in DN hydrogel systems was shown to increase the tensile strength. The GG is used to increase the overall biocompatibility of the hydrogel, whereas PEGDMA is used to overcome the brittle nature of the GG in polymeric networks [240]. Studies also suggest that incorporating silica into an acrylamide-based DN hydrogel will improve the mechanical properties, because the silica particles create a third layer of interaction with the primary polymeric network [241]. This third layer forms a base for enhanced interactions between the polymeric networks, resulting in the strengthening of the DN hydrogel. In the context of higher mechanical stability, research has also been done into the incorporation of calcium sulfate into hydrogel systems to increase and strengthen ionic interactions. Calcium sulfate is used in an attempt to enhance the ionic bonding in the secondary network of the acrylamide–agarose DN hydrogel system [241]. Compared to the weak, conventional single-network hydrogels, DN hydrogels have been found to display superior properties: increased mechanical strength and better flexibility [1,231,242,243]. The first network of the DN hydrogel is rigid and brittle, formed by highly covalent cross-linking by physical interactions. The second network of the DN hydrogel is loosely cross-linked, resulting in a neutral, soft, and ductile network, which is formed by chemical interactions upon photoinitiation [239] (Figure 11).

## 5. Mechanical Properties: Robust, Tougher, and Stretchable Hydrogels

With the development of rapid prototyping tools for building more biocompatible and biodegradable soft robots [244,245,246], the demand to design/synthesize tougher, robust hydrogels has also increased greatly in the recent years [3,25,247,248]. A standard hydrogel is required to contain a hydrophilic material for water absorption and have a hydrophobic content to increase the mechanical toughness. For example, a general hydrogel matrix is brittle and unstable (fracture energy in the range of 10 J m${}^{-2}$) [249], but recent reports have demonstrated a giant leap in hydrogel performances (fracture energy in the range of 100–9000 J m${}^{-2}$) [3,91,250,251,252,253,254,255,256,257,258,259]. Concerning the regime of the fracture energy density of highly robust, stretchable hydrogels, we recently reported a DN hydrogel-based biomedical soft robot that demonstrated a tensile strain of 851% and a maximum tensile strength of 0.273 MPa. Extensive efforts have been demonstrated by the Zhigang Suo’s Group (Harvard University, Cambridge, MA, USA) (see [259,260,261,262,263,264,265,266,267,268,269]) and Xuanhe Zhao’s Group (Massachusetts Institute of Technology, Cambridge, MA, USA) (see [6,209,270,271,272,273,274,275,276,277,278,279]) to make even more stable, flexible, and robust hydrogel matrices for a vast range of potential applications.

Some important properties to be considered while designing hydrogels are the elastic modulus, the fracture strength, Poisson’s ratio, and the stiffness. Being gels, hydrogels have a relatively lower elastic modulus (1–100 kPa range) with respect to metals and ceramics. Therefore, they easily deform or bend when exposed to moderate forces. Similarly, they have a low fracture strength because of their semisolid nature and cannot be designed to work as supports or in structural applications. Poisson’s ratio for a hydrogel network system determines various characteristics of the hydrogel’s environmental reactions, such as volume phase transitions, swelling kinetics, the bending of polyelectrolyte gels when placed in an electric field, and the formation of gel surface patterns. By default, Poisson’s ratio is assumed to be 0.5, but it can vary within a small range. Additionally, the shear modulus of hydrogels decreases as Poisson’s ratio is increased up to 0.5. The stiffness of the hydrogels should be minimized while increasing the toughness via reinforcing or composite formation; the use of hydrogels in actuators and sensors is to facilitate bending, swelling, and twisting motions, and hence a greater stiffness will become a serious hindrance for the necessary shape changes.

**Figure 12:**
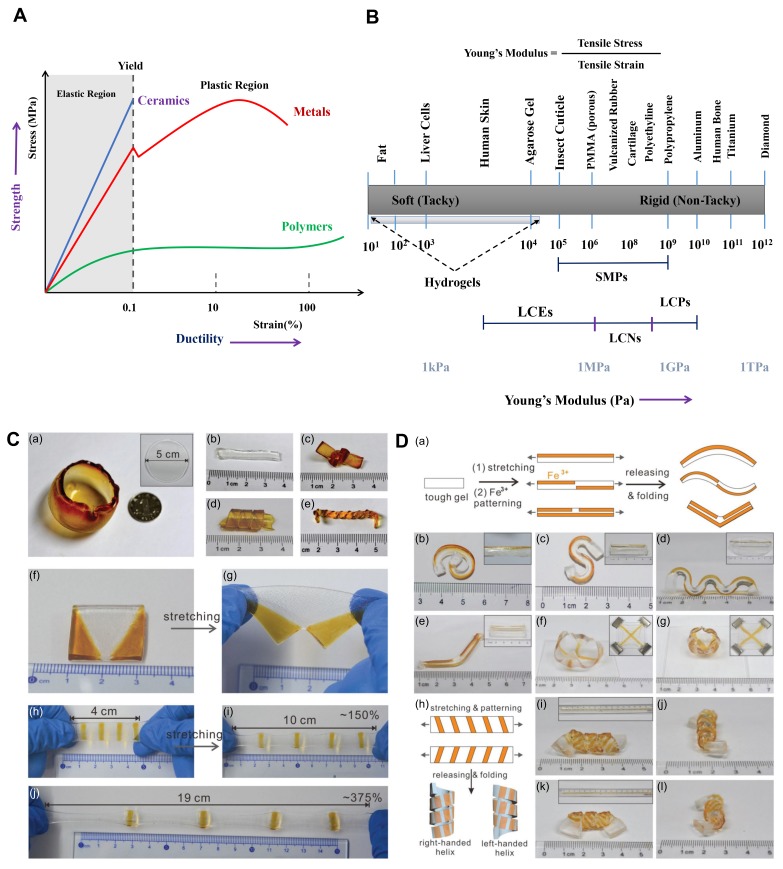
Steps in making robust, tougher, and stretchable hydrogels and their mechanical properties. (**A**) The transition from soft to rigid materials. (**B**) Young’s modulus of various materials. Biocompatible soft hydrogel stiffness fits in the cellular and human skin zone. LCEs: Liquid-crystal polymer elastomers; LCNs: Liquid-crystal polymer networks; LCPs: Liquid-crystal polymers; SMPs: Shape-memory polymers. (**C**) An example of morphing of various “frozen” shapes of tough gels. (**D**) Three-dimensional folding of the Fe${}^{3+}$ ion-patterned Ca–alginate/polyacrylamide (PAAm) tough hydrogels. Adapted with permission from [305]. Copyright 2017, The Royal Society of Chemistry.

To answer the fundamental question of how to achieve a soft hydrogel and yet retain a highly stable and tough mechanical configuration [44,231,280,281,282,283,284,285,286], Xuanhe Zhao presented three distinct design parameters to be taken into consideration: (i) the material should be highly stretchable with increased mechanical dissipation; (ii) the stiffening and fracture of polymer chains should take place simultaneously during failure, resulting in increased fracture strength; (iii) a lower flaw-sensitive length scale should be used, that is, lowering the size of flaws (cracks, cavities, etc.) and increasing the fracture strength [287]. To validate principle (i) presented here [287], the total fracture toughness of the hydrogel layers can be quantified as per the following equation:

(1) $$\Gamma =\Gamma_{0}+\Gamma_{D},$$

where $\Gamma_{0}$ defines the intrinsic fracture stiffness and $\Gamma_{D}$ illustrates the passive zone dissipation [287]. The total energy loss of the hydrogel polymer during the repetitive loading/unloading cycle can also be manifested through its hysteresis loss [288]. For simultaneous fracture and stiffening in the polymer network while under stress, as described in principle (ii), the fracture strength can be quantified in terms of nominal stress as follows [251]:

(2) $$s_{ideal}=N\times \sqrt{n}\times b\times f,$$

where *N* is defined to be the number of chains in the polymer network per unit volume, *n* represents the Kuhn monomers per chain, *b* denotes the length of a Kuhn monomer, and *f* represents the rupture force of the polymer network [289]. Finally, to quantify the flaw-sensitive length scale of a polymer as given in principle (iii), it can be harnessed as the following critical length scale [290,291]:

(3) $$a_{c}=\beta \frac{\Gamma }{W_{c}},$$

where $W_{c}$ defines the work done to rupture a polymer material without any flaw and β is a dimensionless entity that accounts for nonlinear, geometrical effects [287]. The oscillation of the active polymer membrane based on soft hydrogels and the deformation of active matrices under continuous loading conditions also depend on the strain energy density function, principally governed by the Young’s modulus [292]. To address this fundamental principle, we hereafter present a spectrum of the Young’s modulus for different materials (soft to hard transition) (Figure 12).

The elastic modulus of a monoionic polymer gel can be produced from the fundamental principle of elastic rubber theory [293], as defined byj

(4) $$G=A\frac{\rho }{\bar{M_{c}}}RT{\left(v_{2}^{0}\right)}^{\frac{2}{3}}{\left(v_{2}\right)}^{\frac{1}{3}};$$

where *A* is defined as a proportionality constant, the value of which is close to 1, ρ represents the density of the polymer matrix, $\bar{M_{c}}$ denotes the mean molecular weight of the cross-linker, and $v_{2}^{0}$ and $v_{2}$ represents the volume fraction of the gel when it is freshly prepared and swollen, respectively [4]. The swelling problem and the drying out of the hydrogel when exposed to the environment are non-trivial threats posed when designing robust actuators and sensors to last long time spans. In this realm, dehydration and preservation kinetics need to be properly calibrated [231], and a thin-layer coating with polydimethylsiloxane, Sylgard${}^{®}$ 184, polyurethane, latex, 3M${}^{™}$ VHB${}^{™}$ tape, Ecoflex${}^{®}$, among others can also help to restrict dehydration [273]. The excessive swelling of hydrogels hinders stretchability, and hence the reduced elastic moduli can be quantified as

(5) $$\frac{G}{G_{0}}={\left(\frac{v_{2}}{v_{2}^{0}}\right)}^{\frac{1}{3}},$$

where $G_{0}$ and *G* represent the initial and swelling elastic moduli of the hydrogel matrices, respectively [4,294,295]. The internal strength (σ) of any hydrogel polymer matrix is dependent on its surface fracture energy (γ), while plastic deformation/void formation, the elastic modulus (*E*), and particularly the crack length (*c*) are formed during loading/unloading cycles [296,297].

(6) $$\sigma ={\left(\frac{2\gamma E}{\pi c}\right)}^{\frac{1}{2}}.$$

Hence, to optimally synthesize a tough, highly stretchable hydrogel polymer, the energy dissipation principle and recovery need to be properly tuned while the hydrogel ionic bonds break during stretching and are reformed after unloading cycles [298]. There are several chemical bonds that interplay in the formation of tough hydrogel polymer matrices, for example, reversible hydrogel bonds [299,300,301], hydrophobic interactions [302,303,304], and many others, as briefly discussed in the following sections.

## 6. Rapid Prototyping Tools: Hydrogel-Based 3D Printing for Biomedical Soft Robots

**Figure 13:**
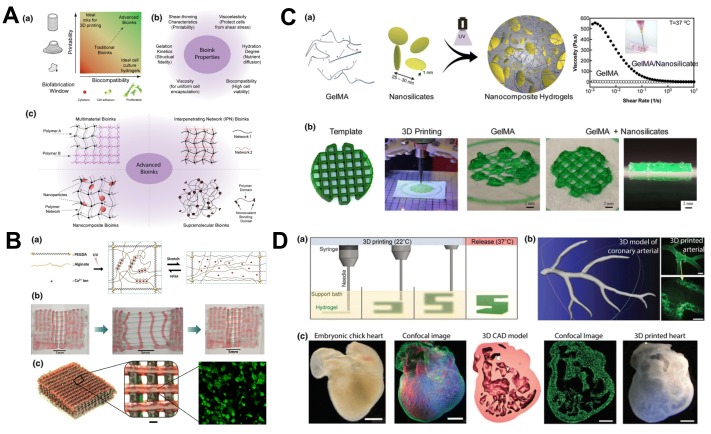
Hydrogel-based 3D printing for artificial organs, and soft biosensors. (**A**) Advanced bioinks for 3D printing, (**B**) interpenetrating network (IPN) bioinks for 3D printing, (**C**) nanoengineered hydrogel-based bioinks for 3D printing, and (**D**) multi-material bioinks for 3D bioprinting using an artificial support bath. Reproduced with permission from [311]. Copyright 2016, Springer Nature Ltd.

Three-dimensional printing refers to processes in which materials are joined or solidified under computer control to create a 3D object. The objects can be of almost any shape or geometry and are generally produced using the digital model data from a 3D model file or an additive manufacturing file (AMF). In tissue engineering and in the manufacture of hydrogel actuators/sensors, hydrogels are used in 3D printing widely; 3D hydrogel printability studies are very important in tissue engineering. Therefore, many reports have been made in the regime of tests on printability and the consistency of properties of hydrogels during the process of gel-based printing [7,306,307]. Hydrogel 3D printing is generally classified into three types: laser-, nozzle-, and inkjet-based printing. In all three cases, a hydrogel reservoir is used from which the hydrogel is infused through any of the above methods, whereas the laser-based printing alone is done with photopolymerization. In one study, the 3D printing of hydrogels for bone and cartilage tissues, which are in high demand in the biomedical field for the replacement of damaged cells and tissues caused by injuries, was studied [308]. Composite hydrogels (such as hyaluronic acid, gelatin, and PEG) are better for use in the manufacturing of tissues and bones than the natural hydrogels because of their enhanced properties, but they make printing and selection for various requirements more difficult [309]. In addition to this is an example in which 3D printing of DN hydrogels was done in a two-step method using a low-cost 3D printer, resulting in higher mechanical properties, such as elastic modulus and compression strength, than those of cartilage [310]. In Figure 13, we show some techniques in 3D printing of complex biological structures and sensors.

In general, it is easier to print stiffer and more hardened hydrogels than soft materials. There was a study in which tough hydrogel composites with varying material properties were 3D printed using an extrusion gradient printing system whereby the mixing of two inks was monitored using software [312,313]. However, there are three main difficulties in printing soft hydrogels: (i) they are semisolid in nature, (ii) they have low viscosity, and (iii) the resulting hydrogel should be minimally stiff to produce soft actuators and sensors. This can lead to the production of hydrogels with a lower resolution and in parts. Even if we try to increase the hydrogel concentration, this can lead to hardened and inflexible hydrogels after printing. Thus, a method was designed to print low-viscosity hydrogels in a single scaffold. The 3D printing was done at a sub-zero temperature at three different pressures (30, 40, and 50 kPa), resulting in lower variation in the strut diameter and increased spatial resolution [314]. Another method for 3D printing was also proposed whereby the hydrogels are 3D printed cryogenically, resulting in a very low stiffness of very few kilopascals, mimicking very soft human tissues such as those of the brain or lungs. The cryogenic setup was established using solid CO${}_{2}$ (dry ice) and an isopropanol thermal conductive bath. This method can also produce hollow structures in soft hydrogels [315]. In Table 5, we illustrate different stimuli-based hydrogel chemical response and their applications.

**Figure 14:**
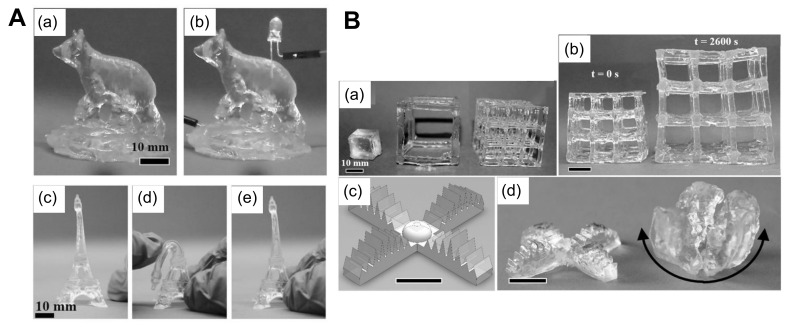
Highly elastic, transparent, and conductive 3D printed ionic composite hydrogels. (**A**) (**a**) 3D printed transparent, conductive ionic composite hydrogel bear, (**b**) a light-emitting diode (LED) lights up when electric potential is applied across the object, and (**c**–**e**) demonstration of resilience of a 3D printed Eiffel tower after repeatable extensive deformation. (**B**) (**a**) Printed ionic composite of different surface area/volume ratios with different swellings, (**b**) a high-surface area/volume ratio ionic composite hydrogel before and after water absorption, (**c**) design of a multi-armed hydrogel gripper, (**d**) before and after swelling in blue dyed water. Reproduced with permission from [326]. Copyright 2017, John Wiley & Sons, Inc.

Three-dimensional printing has high potential in the future for the production of hydrogel sensors and actuators, as concluded from the above points, as there are constant improvements being made to produce very soft hydrogels. For example, researchers performed a study on the humidity-driven actuation of hydrogels produced by 3D printing in a two-photon polymerization where the humidity-driven swelling was controlled by adjusting the cross-linking density of voxels in the microstructure [322]. In another study, the 3D printing of soft actuators was performed for numerous external stimuli and controls, producing customized actuators for real-life applications [142]. An example of the production of a stimuli-responsive hydrogel for actuation is a temperature-responsive hydrogel that was micro-3D printed by projection microstereolithography, a method in which the rapid photopolymerization of a layer is done by a flash of UV light at the microscale resolution [323]. Additionally, a study was performed on the design and fabrication of a skeleta muscle-powered biomachine controlled via external signaling on the basis of a 3D printing-based biofabrication system [324]. On the other hand, the 3D printing of hydrogel sensors is also becoming commonly used. All types of sensors, such as biosensors, chemical sensors, and physical sensors, are prepared by directly printing the sensing components or by printing moulds or platforms for the sensors [325]. Additionally, skin-like sensors were produced by the 3D printing of thermo-responsive hydrogels [324]. Recently, researchers at MIT have used 3D printing to create soft, nearly invisible hydrogel robots, which can be useful in underwater applications [6]. In Figure 14, we show different textures and morphologies for 3D printed models with highly compliant hydrogel composite structures.

## 7. Self-Healing Hydrogels for Soft Robotics

Self-healing refers to the immediate formation of new bonds when old bonds are broken between material molecules. This property is also inspired from nature; for example, lizards can grow/heal their tails back when they are cut [2]. Hydrogels have this ability, as they are composed of hydrophilic polymers as well as water. This makes synthetic hydrogels very similar to biological tissues, and their regenerative features are similar under certain exposures [327]. Extending this ability, they find applications such as “elastic second skins” [328], which are artificial synthetic skins used for providing the look of youthful skin free from wrinkles. Additionally, this type of hydrogel will be useful for artificially intelligent skins and other enhancements for artificial intelligence in the near future [329]. The material used here is a silicone-based polymer that could be applied on the skin as a transparent/invisible coating [50]. In addition, these hydrogels can be strengthened through hydrophobic interactions via free-radical cross-linking polymerization [256]. These transparent hydrogels can be synthesized by the micellar copolymerization of acrylamide (AAm) with 2 mol % C18 in the presence of NaCl concentrations (between 0.3 and 0.8 M) because of the complete solubilization of C18 [301,330].

Furthermore, these hydrogels can be enhanced by “anti-biofouling”. Biofouling refers to microorganisms or proteins becoming attached easily to hydrogels and hence obstructing the circulation of biomolecules, leading to inflammation. In this study, an injectable self-healing hydrogel triblock copolymer poly[(*N*-isopropylacrylamide)-co-(*N*-3,4-dihydroxyphenethyl acrylamine)]-*b*-poly(ethylene oxide)-*b*-poly[(*N*-isopropylacrylamide)-co-(*N*-3,4-dihydroxyphenethyl acrylamine)] (DNODN) with anti-biofouling properties was made in a metal-free aqueous environment [331]. Additionally, it could be made more stretchable with GO/poly(acryloyl-6-aminocaproic acid) (PAACA) composite and the speed of healing could be increased by balancing hydrophilic and hydrophobic forces [332]. In another study, shape-memory alloy (SMA) hydrogels underwent an elastic deformation, being temporarily shaped by reversible covalent or physical cross-links, resulting in a temporary shape [284]. However, the material could reverse to the original shape when a suitable stimulus was applied. This is useful for the growing/diminishing of shapes in the required hydrogel as per the external stimuli [333,334,335].

**Figure 15:**
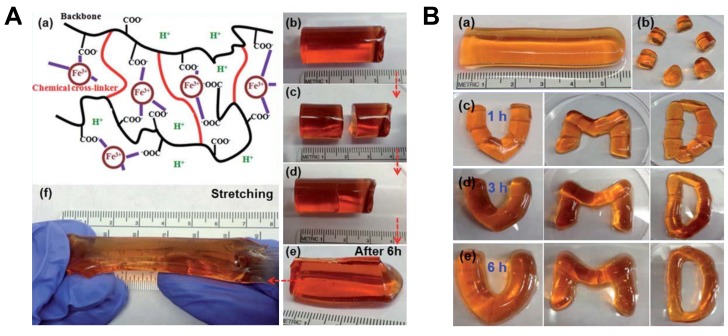
Self-healing poly(acrylic acid) (PAAc) hydrogels for potential biorobotic applications. (**A**) (**a**) Schematic illustration of the structure of the self-healing hydrogel. (**b**–**e**) Self-healing of a cylindrical hydrogel (Entry 3) at room temperature: (**b**) the original hydrogel, (**c**) the hydrogel after being cut, (**d**) the hydrogel after the two parts were brought into contact with each other, (**e**) the hydrogel after healing for 6 h. (**f**) Stretching the self-healed hydrogel up to 200%. (**B**) The use of the self-healing nature of the hydrogel to produce complex architectures. (**a**) The original hydrogel. (**b**) The hydrogel after being cut. (**c–e**) The hydrogel segments arranged in the form of letters: U, M, and D, after (**c**) 1, (**d**) 3, and (**e**) 6 h of self-healing. Reproduced with permission from [337]. Copyright 2013, The Royal Society of Chemistry.

Thus, there are many characteristics that are to be considered when designing the skin (safety, ability to readily spread on skin, adherence to skin, and mechanical properties such as those of normal skin) [336]. The cross-linked polymer layer (XPL) is used as the material here; it depends on elastic recoil, flexibility, and elongation. Adding fumed silica increases the mechanical toughness but also increases the viscosity of the reactive polymer blend (RPB) [328]. Generally, synthetic hydrogels are very weak and do not have the property of self-healing. Their mechanical strength can be increased by free-radical cross-linking polymerization. They are prepared by copolymerization of the hydrophilic monomer AAm with a small amount of a hydrophobic comonomer through a micellar polymerization process. Transparent gels were obtained in the range of Csalt between 0.3 and 0.8 M as a result of the complete solubilization of C18 in the sodium dodecyl sulfate (SDS) micelles. At higher salt concentration, although the initial reaction solutions were transparent, opaque gels or solutions (Csaltg3.0) were obtained after the polymerization process, suggesting that a phase separation occurred during the reactions as a result of the aggregation of micelles. Here, salt concentrations in the reaction solutions were limited to below 1 M NaCl to obtain transparent hydrogels [256]. Figure 15 shows the timing for the hydrogel to completely heal and its mechanical stability.

To enable shape-switching of hydrophilic polymer networks the shape-memory effect (SME) involves an elastic deformation (programming) of samples, which are temporarily fixed by reversible covalent or physical cross-links, resulting in a temporary shape. The material can reverse to the original shape when the application of a suitable stimulus affects these molecular switches. The healing rate can be increased by balancing the hydrophilic and hydrophobic forces. Self-healing is generally seen in linear polymers, supramolecular networks, dendrimer–clay systems, metal ion–polymer systems, and multi-component systems. Differently coated surfaces with acryloyl-6-aminocaproic acid (A6ACA) hydrogels were applied and then tested by deliberately damaging and cracking them, which resulted in healing within seconds upon exposure to low pH buffers; positively, they required only initial contact [301].

## 8. Conclusions and Future Challenges

In this review article, we have briefly introduced hydrogels, their structures, and their usage in soft robotics for biomedical applications in the form of actuators and sensors, mainly from a structural mechanics point of view. From the data we have collected and critically analyzed, we can conclude with respect to various sources that hydrogels will become a demanding and burgeoning material in soft robotics in the near future, despite that their usage and chemical processing are rudimentary at present. Superior to the conventional materials, such as PDMS and silicone elastomers, because of their hydrophilic nature, swelling/bending capabilities, high biocompatibility, self-healing capabilities, and stimuli-responsiveness to numerous environmental factors, hydrogels are becoming more preferable for use in actuating and sensing mechanisms for bioapplications than ever before [287]. Furthermore, the possibility and resources available to produce mechanically stable, hybrid composites combining other polymers is also another merit for their use. The important stimuli-responsive actuators and sensors in the current research are discussed, along with the 3D printing of hydrogels and the challenges facing its application. Adding to this, a previous limitation of hydrogel fabrication was the slow multi-step free-radical polymerization process, which also makes it difficult to control the molar ratio of monomers, although it has been overcome recently with the introduction of better methods, such as the new one-pot synthesis procedure developed by Chen et al. [239]. Although many of the promises depicted here can potentially be applied in soft robotics and the associated biomedical realm, one key limitation is the spatial gel inhomogeneity present in hydrogels (inhomogeneous cross-link density distribution), which reduces the optical clarity and strength. The other limitations to be overcome in designing hydrogel robots are the high cost, non-adherence, and poor mechanical strength due to their high water content; therefore, hydrogels are currently used only as coatings and are difficult to sterilize. In conclusion, although this review paper discusses avenues for hydrogels’ potential and challenges from a mechanical stability standpoint, there are still many collaborative cross-departmental efforts to be made for this technology to mature in addressing the 21st century biomedical challenges. 

## Figures and Tables

**Table 1:** Some selected key comparisons between hydrogel and silicone elastomer properties.

Hydrogels	Silicone Elastomers
Excellent water-absorbing properties	Excellent elastic properties
Volume changes with external stimuli	Less responsive to external stimuli
Preferred as biomaterials, e.g., dressing wounds	Chemically inert and more toxic
Absorb body fluids; very soft and bendable	Good absorbers of gases
Excellent transport properties, e.g., controlling drugs and nutrient release	Generally hydrophobic; cannot retain fluids
Semi-solid/liquid state; better relaxation behavior	Mixed with hydrogels give better hydrophilic properties
Mostly water; hence better fire-retardant skin-based devices	Better oxygen permeability and transport
Gelatin is hemostatic; hence faster wound healing	Highly viscous and hydrophobic
Better mechanical strength in composite, better self-healing time, etc.	Less responsive
Resist protein adsorption; good wettability; can be used in soft contact lenses	Less likely to be used
Adaptable to small, delicate structures	Inability to adapt to small scale
Relatively complex preparation protocol	Ease of preparation

**Table 2:** Some key comparisons between different types of actuators. Here, we portray a rough estimate of the actuation properties, which may differ in practice with actuator size and heating power. Adapted with permission from [53]. Copyright 2009, Springer.

Actuator	Energy Density (J cm${}^{-3}$)	Elongation (%)	Pressure (MPa)	Response Time (ms)
Solenoids	0.025	50	0.1	5
Piezo-actuators	0.05	0.2	110	0.5
Magnetostrictive	0.025	0.2	70	0.4
Electrostrictive	0.17	32	2	2
Shape-memory alloy	10	8	900	300
**Hydrogels**	**0.35**	**90**	**4**	**300**
Electrochemical	0.14	50	25	16
Electrostatic	0.0015	50	0.03	0.003
Muscle	0.59	70	1.18	0.03

**Table 3:** Advantages and limitations of hydrogel-based soft actuators for different stimuli. Adapted with permission from [142]. Copyright 2016, IEEE.

Stimulus	Advantages	Limitations	Reference
Thermal	Ease of operation	Slow response	[143,144]
Electrical	Faster response	Electrolyte layer formation	[145,146]
pH	Faster response, reversible volume change	Complex pH solution formation	[147,148]
Magnetic	Wireless, remote-controlled	Brittle structure, difficult to print	[147,149]
Light	Wireless, remote-controlled	Difficult to control and penetrate	[80]

**Table 4:** Strategies for small biomolecule detection using hydrogel-based glucose biosensors.

Hydrogel	Transduction Strategy	Specification	Reference
Polyaniline	Electrochemical	RT: 3 s; LR: 0.01–8 mM	[179]
Polyaniline–PEG	Electrochemical	-	[180]
PEG	Optical	RT: 10 min; LR: 0–600 mg/dL${}^{-1}$	[166]
PVA–vinyl pyridine	Electrochemical	RT: 11 s	[181]
Chitosan	Electrochemical	RT: 7 s; LR: 5 μM to 2.5 nM	[182]
Chitosan–graphene oxide	Electrochemical	LR: 0.02–6.78 mM	[183]
Polypyrrole	Electrochemical	LR: up to 15 mM	[184]
PEG (injectable)	Optical	RT: 11 min; LR: up to 370 mg dL${}^{-1}$	[185]
Polyvinylpyrrolidone	Optical	-	[186]
Alginate	Optical	LR: 2.6–350 mg/dL${}^{-1}$	[187]
HEMA	Electrochemical	LR: 10 μM to 40 mM	[188]

HEMA: Hydroxyethyl methacrylate; LR: Linear range; PEG: Polyethylene glycol; PVA: Poly(vinyl alcohol); RT: Response time [162].

**Table 5:** Hydrogel-based 3D printing with different stimuli. Adapted with permission from [142]. Copyright 2016, IEEE.

Chemicals	Stimulus	Reference
Alginate/poly(*N*-isopropylacrylamide) (pNIPAM) Polycaprolactone Alginate/poly(acrylamideionic covalent entanglement) (Alg/PAAmICE) Pluronic F127 Gelatinmethacryloyl (GelMa)	Thermal	[143,147,316,317,318]
Poly(2-acrylamido-2-methylpropanesulfonic acid) (PAMPS) 4-Hydroxybutyl acrylate (4-HBA) Acrylamide (AAm)/sodium acrylate (NaAc)	Electrical	[52,145,146]
Poly(methacrylic acid) (PMAA) Collagen Keratin Polyacrylic acid (PAA)	pH	[147,148]
Magnetic nanoparticles such as Fe${}_{2}$O${}_{3}$, Fe${}_{3}$O${}_{4}$, FePt, and CoFe${}_{2}$O${}_{4}$ in polymers such as poly(2-hydroxyethyl methacrylate) (pHEMA)	Magnetic	[110,147]
Gellan gum methacrylate (GGMA) Graphene oxide (GO)/pNIPAM	Light	[319,320,321]
